# Stakeholder analysis of the Programme for Improving Mental health carE (PRIME): baseline findings

**DOI:** 10.1186/s13033-015-0020-z

**Published:** 2015-07-08

**Authors:** Amit Makan, Abebaw Fekadu, Vaibhav Murhar, Nagendra Luitel, Tasneem Kathree, Joshua Ssebunya, Crick Lund

**Affiliations:** Department of Psychiatry and Mental Health, Alan J Flisher Centre for Public Mental Health, University of Cape Town, 46 Sawkins Road, Rondebosch, 7700 Cape Town South Africa; Department of Psychiatry, College of Health Sciences, School of Medicine, Addis Ababa University, PO Box 9086, Addis Ababa, Ethiopia; Department of Psychological Medicine, King’s College London, Institute of Psychiatry, Centre for Affective Disorders and Affective Disorders Research Group, London, UK; Public Health Foundation of India, Sangath House, House Number 6, Rishi Nagar, Char Imli, Bhopal, 462016 Madhya Pradesh India; Transcultural Psychosocial Organization Nepal, Baluwatar, Box 8974, Kathmandu, GPO Nepal; School of Psychology, University of KwaZulu-Natal, Howard College Campus, Durban, 4000 South Africa; Butabika National Mental Hospital, Kampala, Uganda

**Keywords:** Stakeholder analysis, Health policy and systems research, Knowledge translation, Research uptake, Mental health

## Abstract

**Background:**

The knowledge generated from evidence-based interventions in mental health systems research is seldom translated into policy and practice in low and middle-income countries (LMIC). Stakeholder analysis is a potentially useful tool in health policy and systems research to improve understanding of policy stakeholders and increase the likelihood of knowledge translation into policy and practice. The aim of this study was to conduct stakeholder analyses in the five countries participating in the Programme for Improving Mental health carE (PRIME); evaluate a template used for cross-country comparison of stakeholder analyses; and assess the utility of stakeholder analysis for future use in mental health policy and systems research in LMIC.

**Methods:**

Using an adapted stakeholder analysis instrument, PRIME country teams in Ethiopia, India, Nepal, South Africa and Uganda identified and characterised stakeholders in relation to the proposed action: scaling-up mental health services. Qualitative content analysis was conducted for stakeholder groups across countries, and a force field analysis was applied to the data.

**Results:**

Stakeholder analysis of PRIME has identified policy makers (WHO, Ministries of Health, non-health sector Ministries and Parliament), donors (DFID UK, DFID country offices and other donor agencies), mental health specialists, the media (national and district) and universities as the most powerful, and most supportive actors for scaling up mental health care in the respective PRIME countries. Force field analysis provided a means of evaluating cross-country stakeholder power and positions, particularly for prioritising potential stakeholder engagement in the programme.

**Conclusion:**

Stakeholder analysis has been helpful as a research uptake management tool to identify targeted and acceptable strategies for stimulating the demand for research amongst knowledge users, including policymakers and practitioners. Implementing these strategies amongst stakeholders at a country level will hopefully reduce the knowledge gap between research and policy, and improve health system outcomes for the programme.

**Electronic supplementary material:**

The online version of this article (doi:10.1186/s13033-015-0020-z) contains supplementary material, which is available to authorized users.

## Background

The use of stakeholder analysis (SHA) as a systematic technique for gathering insights relating to a proposed action or reform is not new, and has commonly been used in business, change management, public policy, health care management and development. SHA gathers these insights by identifying, categorising and analysing individuals or groups that are likely to have a ‘stake’ (be affected by, or have an interest in) a proposed action [1–3].

More recently, the utility of this approach has been reiterated amongst scholars of Health Policy and Systems Research (HPSR) [4–6]. HPSR has evolved into an interdisciplinary field encompassing the policy realm, acknowledging the interconnections between policy and health systems, and highlighting the social and political nature of healthcare [5]. SHA has been developed to better understand stakeholder power and positions around specific new policies or actions, and assess the likely implications for the acceptability of new policies or interventions. However, published research regarding its use or how to perform such analyses within the context of HPSR has been limited [4, 7].

Health systems often fail to effectively implement evidence-based public health interventions, particularly in Low- and Middle-Income Countries (LMIC). This is due to poor knowledge translation, over-emphasising the production (supply) of research, rather than stimulating its consumption (demand) [8]. Defined by the Canadian Institutes of Health Research, knowledge translation is a “dynamic and iterative process that includes the synthesis, dissemination, exchange and ethically sound application of knowledge to improve health, provide more effective health services and products, and strengthen the health care system [9]”.

In order to understand the ‘needs’ of knowledge users and minimise poor knowledge translation, SHA has been applied as a technique to firstly identify the stakeholders (many of whom are likely to be knowledge users), and to gather insights into their position vis-a-vis the proposed action of a mental health research programme. Such insights will likely point to strategies for stimulating the demand for research amongst knowledge users, and hence, minimise the knowledge gap between research and policy.

Mental health is no exception to poor knowledge translation, and the failure of health systems implementing evidence-based interventions. This is supported by the assertion of global mental health scholars that mental health does not receive the policy attention expected of it globally [10–13].

The primary aim of this study is to document the value of SHA as a technique for identifying and characterising support for the proposed action. In addition, the study aims to identify the level of involvement of stakeholders within the PRogramme for Improving Mental health carE (PRIME).

PRIME aims to generate health systems research on the best ways to integrate and scale up mental health into maternal and primary health care systems in LMIC, and to stimulate demand for this research. PRIME does this by advocating for evidence-based decision-making, and by promoting the uptake of its implementation research amongst knowledge users, including health and related sector policymakers and practitioners, through a dedicated ‘Research Uptake Strategy’ [14]. PRIME is piloting the scale-up of mental health services in one District in each country: Sodo in *Ethiopia*, Sehore in *India*, Chitwan in *Nepal*, Dr Kenneth Kaunda in *South Africa* and Kamuli in *Uganda* [15].

In terms of SHA, the ‘proposed action’ identified is linked to the goal of PRIME, which is to scale-up mental health services in the districts of the five LMIC. Until the advent of this initiative, little was known about PRIME stakeholders, and their engagement with mental health policy and systems research. The SHA technique is intended to enable PRIME to identify and understand stakeholder power and positions, and assess the likely implications for the acceptability of the proposed action. Furthermore, this paper aims to add further knowledge about the method of SHA by sharing the experiences of its application in the field of mental health.

## Methods

The study design is that of a qualitative stakeholder analysis. PRIME’s research programme consists of three phases over a 6-year period: Inception Phase, Implementation Phase and Scaling Up Phase [14]. As part of the Inception Phase, formative research was conducted including literature reviews, a situation analysis of mental health systems [15], in-depth interviews and focus group discussions with stakeholders [16] and Theory of Change (ToC) workshops [17]. During the Inception Phase, the following systematic steps were followed in order to collect the cross-country data required for qualitative content analysis of stakeholder perceptions.

### Formulation of PRIME’s research uptake strategy

**Figure 1:**
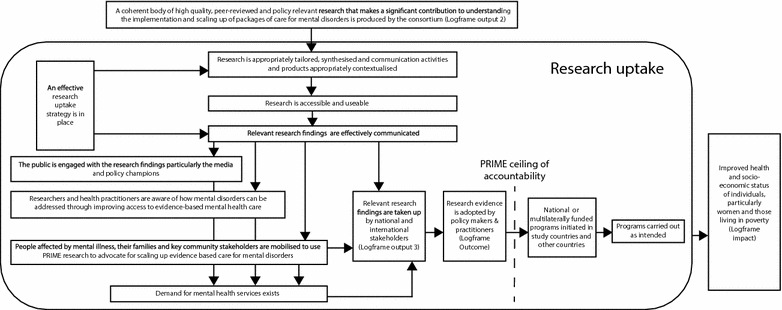
PRIME theory of change for a research uptake strategy. The figure highlights the role of PRIME’s Research Uptake Strategy in the context of its Theory of Change map.

A research uptake strategy was formulated as part of PRIME’s Theory of Change (ToC) framework describing the pathway between research evidence and policy impact. The purpose of this strategy was to ensure that the research evidence produced by PRIME would be translated into policy and practice (illustrated in Figure 1). The Development Research Uptake in Sub-Saharan Africa (DRUSSA) programme describes research uptake as a management process “working with scientific research that has both a traditional focus on building and disseminating the bodies of knowledge created in the academic domains, and a newer and wider focus on maximising the conditions for the application of these bodies of knowledge to achieve outcomes that have a developmental impact [18]”.

The research uptake strategy took into consideration the three levels of the health system that PRIME is integrating mental health into primary health care: health service organisation, health facility and community levels [14]. The initial research uptake strategy was developed in consultation with 15 PRIME country and cross-country partners. Given the dynamic and often fickle nature of the pathway from research to policy, the strategy is understood by partners as a living document, open to adaptation as circumstances change.

### Identifying stakeholders

The research uptake strategy identified a broad range of groups that may have a ‘stake’ in the objectives of the programme, and who could influence the translation of research findings into policy and practice. Specific stakeholders within groups were also identified at a country level (e.g. Ministry of Health in the category for ‘Policy Makers’).

Traversing the three levels of health system, these groups included:

1. Health practitioners (mental health specialists, general primary health care workers including doctors and nurses, and community health workers);
2. Persons affected by mental illness including those with psychosocial disabilities, their families, carers and service user groups;
3. Civil society organisations including Non-Governmental Organisations (NGOs), Community Based Organisations (CBOs) and Faith Based Organisations (FBOs);
4. The media at all levels (international, regional, national, state and district);
5. Donors including DFID UK, DFID regional or country offices, and other funding agencies; and
6. Policy makers, including WHO and Ministries of Health, other intersecting Ministries or government departments (such as social development, economic development, correctional services, police services, peace and reconciliation) and parliamentary committees such as health, and related sector committees.

Although translating the research findings into policy and practice is one of the goals of the programme, the rationale for including a diverse set of stakeholders is based on the premise that each has a significant role to perform in the knowledge translation process by stimulating the demand for the evidence-based scale up of mental health services [11, 19].

### Characterising stakeholders by country

Having identified the broad stakeholder groupings, a comprehensive stakeholder analysis instrument was constructed using Varvasovszky and Brugha’s ‘stakeholder characteristics’ table as a framework [20]. The table recorded stakeholders’ interest, influence, position and impact in relation to the issue (integrating and scaling-up mental health care) for each country.

In order to minimise the potential for bias from individual analysis and considering the feasibility of various data collection options across five countries, PRIME researchers and project co-ordinators from country teams were identified to lead the completion of stakeholder analysis tables as a component of the country-level research uptake strategy across the consortium. Stakeholder tables were completed based on local knowledge at the time and initial stakeholder engagements early in the programme. The knowledge was informed by a wide range of engagements that took place during PRIME’s formative research process (introduced early in the methods section above), including situation analysis, theory of change workshops, in-depth interviews and focus groups. The findings of this formative research informed the PRIME partners’ assessments of the various stakeholders included in the stakeholder analysis. The interviews in the formative research included questions about various stakeholders’ perceptions of mental health, the feasibility and acceptability of integrating mental health into primary care and the packages of care that should be included in the PRIME mental health care plans [16, 17, 21].

A participant from the country teams was identified to lead the completion of the stakeholder analysis instrument. This was done through group brainstorming and by consensus, with core team members contributing to the characterisation of stakeholders. The lead participants included the PRIME Principal Investigator in Ethiopia (psychiatrist), and project coordinators in India, Nepal, South Africa and Uganda (either researchers or research psychologists). Additional team members contributing to the characterisation included principal investigators, site coordinators and research assistants.

### Analysing stakeholders across countries

A cross-country stakeholder analysis table was developed for each stakeholder group, synthesising five country tables of stakeholder characteristics (see Additional file 1: Table S1, Additional file 2: Table S2, Additional file 3: Table S3, Additional file 4: Table S4, Additional file 5: Table S5, Additional file 6: Table S6, Additional file 7: Table S7). This facilitated cross-country comparisons by stakeholder groups. Qualitative content analysis was conducted across countries based on the following variables identified by Varvasovszky and Brugha [20]:

- Involvement in the issue/proposed action (described for each stakeholder);
- Interest in the issue/goal (coded low, medium or high)
- Influence/power over the issue/goal (coded low, medium or high)
- Position regarding the issue/goal (coded supportive, opposed or neutral/non-mobilised)
- Potential impact of the proposed action on the stakeholder (coded low, medium or high)

### Force field analysis

Given the array of stakeholders identified across countries and the vast potential engagement opportunities that the analysis yielded, a mechanism was necessary to summarise stakeholder positions for higher-level analysis. Traditionally used in the field of change management, force field analysis has been suggested by scholars of the stakeholder analysis technique as a useful means of summarising stakeholder analyses. A force field analysis, adapted from a hybrid of approaches from Varvasovszky and Brugha [20] and Gilson et al [4], was applied to the cross country stakeholder analyses.

### Ethical issues

Ethics approval for PRIME was obtained by the institution leading PRIME (Human Research Ethics Committee of the Faculty of Health Sciences, University of Cape Town, UCT HREC Ref 419/2011) and the respective institutions linked to country investigators (Addis Ababa University in Ethiopia, Sangath in India, Nepal Health Research Foundation, University of KwaZulu-Natal in South Africa, Makerere University/Butabika Hospital in Uganda). In accordance with these ethical requirements, the identities of specific stakeholders were kept confidential by country teams.

## Results

### Cross-country analyses by stakeholder groups

Five country teams identified specific stakeholders by group, and tabulated their characteristics in relation to the proposed action: scaling-up mental health care. Cross-country stakeholder analyses of policy makers (Additional file 1: Table S1), donors (Additional file 2: Table S2), health practitioners (Additional file 3: Table S3), persons affected by mental illness (Additional file 4: Table S4), civil society (Additional file 5: Table S5), the media (Additional file 6: Table S6) and academics (Additional file 7: Table S7) are analysed presented at country and cross-country levels (see Additional file 1: Table S1, Additional file 2: Table S2, Additional file 3: Table S3, Additional file 4: Table S4, Additional file 5: Table S5, Additional file 6: Table S6, Additional file 7: Table S7) and described below.

### Policy makers

*PRIME Ethiopia* identified the high level of interest and support of the WHO regional office and the Federal Ministry of Health. Other sectors such as the Ministry of Social and Labour Affairs were reported to be supportive, although with lower levels of interest. Amongst other democratic institutions, Parliament was reported to be supportive given the personal interest of an influential Member of Parliament, although the team reported that greater efforts were necessary for engaging with Parliament.

Given the collaboration of the *PRIME Indian* team with the WHO in implementing the mental health Gap Action Programme (mhGAP), the WHO regional office was reported to have high levels of interest and support. Despite the District (Madhya Pradesh) Department of Public Health and Family Welfare being a collaborator in PRIME consortium, it was reported that the interest in the issue was mixed given that some senior level policy makers may have had low levels of interest, especially considering other priority programmes such as Reproductive and Child Health, Family Planning and HIV/AIDS. Other stakeholders identified as having a medium level of interest in the issue included the Ministry of Health and Family Welfare, and National AIDS Control Organisation (national level), Department of Medical Education, the State Mental Health Authority and State AIDS Control Society in Madhya Pradesh (district level). Amongst these stakeholders, particularly those believed to have a medium impact on the issue, opportunities to mobilise them were identified. Other opportunities for mobilisation identified include stakeholders with a high influence, high impact but low interest, such as the Madhya Pradesh Legislative Assembly, and Panchayati Raj Institutions (local self-governing bodies).

The *PRIME Nepal* team identified the Primary Health Care revitalisation department as having a high interest in the issue, and being supportive of the issue as an active collaborator with PRIME. Other non-health sectors were also identified as having a high interest, specifically the Ministry of Women, Children and Social Welfare; and the Ministry of Peace and Reconstruction, and opportunities were identified to mobilise these Ministries. Although supportive, the WHO country office was believed to have a medium interest, whilst the Ministry of Health and Population was believed to have a low interest due to, in both cases, the prioritisation of other issues. Other democratic institutions, such as Parliament, have not been explored as yet given Nepal’s political environment.

*PRIME South Africa* regarded policy stakeholders, including WHO, Department of Health (national, provincial and district) and other government departments such as social development, as being supportive of the issue. However, it was believed that the stakeholders have medium levels of interest given that it is not a priority. Furthermore, the team indicated that the interest in mental health exists in relation to priority health issues such as HIV/AIDS and maternal health.

*PRIME Uganda* identified the WHO country office and Ministry of Health as having high levels of interest, and as being highly supportive of the issue. Despite low levels of interest, it was believed that other non-health sector departments and democratic institutions such as Parliament had medium–high levels of influence. Opportunities to mobilise these sectors were identified.

### Donors

All countries believed DFID UK to be supportive of the issue, given their funding of the consortium. PRIME in *Ethiopia* and *Nepal* believed regional or country DFID offices to have high levels of interest in the issue, whilst *India* and *South Africa* believed there to be medium levels of interest amongst DFID country offices given the competing priorities of other Research Programme Consortia (RPCs). *Uganda* predicted a low level of interest amongst DFID country offices. Most countries, with the exception of *India*, report medium levels of interest amongst other donors or development agencies.

### Health practitioners

Whilst *Ethiopia* regarded mental health specialists, particularly psychiatrists, as having high levels of interest for the issue, it regarded PHC and community health workers as having medium levels of interest. Despite the support identified, Ethiopia believed that further engagement was needed with mental health specialists and particularly amongst PHC workers, as their involvement is crucial for meeting PRIME’s objectives.

Although *PRIME India* did not mobilise support from mental health specialists at that stage, PHC workers (medical officers and nurses/midwives), community health workers and voluntary workers, were all regarded as having medium levels of interest in the issue. This is due to the fact that they are overburdened, without having much time to devote to things beyond their scope of work, as per the priorities set by the District Health Administration. Voluntary health workers known as Accredited Social Health Activists (ASHA) who are responsible for psychosocial education at the community level were identified as having high levels of influence on the issue, particularly from the perspective of mental health stigma reduction.

In *Nepal*, although health practitioners (PHC workers, community and voluntary health workers) were thought to be supportive, medium levels of interest were registered due to them being overburdened. In the case of mental health specialists, the lower interest was also explained by their presence mostly in urban areas. It was believed that the issue of scaling up mental health services will have a high impact on PHC workers (doctors, nurses and midwives), given the new roles that they will be expected to perform.

*South African* health practitioners were understood to have medium levels of interest, given that support at a provincial level was being nurtured at the time. Nevertheless, the level of impact was understood to be high given the political will of the government at the national level, and the fact that implementation of policy and legislation rests with the provinces and districts.

*Uganda* registered high levels of interest and support amongst mental health specialists, however, recognised that greater support and interest needs to be garnered from PHC and CHWs.

### Persons affected by mental illness

*Ethiopia* believed there to be high levels of interest and influence amongst mental health service user groups, people with psychosocial disabilities, and their families or carers. Although service user groups (such as the Mental Health Society, Ethiopia) were supportive of the issue, their level of influence was regarded as low given the small number of members. Opportunities were identified for mobilising people with psychosocial disabilities and their families or carers, such as their inclusion in Community Advisory Boards (CABs). Based on previous projects, these persons affected by mental illness were expected to be supportive of the issue.

*India* recorded medium levels of interest in the issue amongst service users, people with psychosocial disabilities, and their families or carers. High levels of influence, and impact of the issue on these sub-groups were recognised by India, as have opportunities to mobilise them.

Despite believing a lower level of influence, *Nepal* recorded high levels of interest and support amongst service user groups, people with psychosocial disabilities, and their families or carers in relation to the issue.

Although not yet mobilised, medium levels of interest were predicted by *South Africa*, pointing to the fact that some affected persons may be uninterested due to apathy, discrimination and stigma and a lack of awareness and education about mental health and their right to health care.

In *Uganda*, families or carers of people with psychosocial disabilities were understood to have low levels of interest, followed by medium interest from people with psychosocial disabilities, followed by high levels of interest from service user groups, which are also believed to be supportive of the issue. Opportunities to mobilise people with psychosocial disabilities, and their families have been recognised.

### Civil society

In *Ethiopia*, international NGOs and FBOs were believed to be supportive of the issue, with medium levels of interest. Some in these groups are represented in PRIME’s Community Advisory Board (CAB). The medium–high influence of FBOs, and their power to raise awareness through anti-stigma campaigns have been recognised. Due to the high potential for influencing communities, opportunities to mobilise CBOs were recorded.

In *India*, low levels of interest in the issue were recorded amongst international NGOs, however, medium to high levels of interest were recorded amongst national NGOs, many of whom were regarded as being supportive. Opportunities to mobilise CBOs, FBOs and international NGOs were documented.

*Nepal* recorded high levels of interest and support amongst national NGOs and CBOs, regarding the issue as having a high impact on these groups given their ability to provide technical support to PHC staff, and advocate for people to seek mental health services. Lower levels of interest and support were recorded amongst FBOs and traditional healers given the fact that this group has been providing a service for persons affected by mental illness. Although supportive, international NGOs were also regarded as having lower levels of interest given the competing priorities.

*South Africa* identified some specific international NGOs (Basic Needs) and national NGOs (South African Federation for Mental Health) as having high levels of interest and support, with opportunities to mobilise CBOs and FBOs.

*Ugandan* civil society was recorded as having low-medium levels of interest in the issue. International NGOs were regarded as being supportive of the issue, whilst the opportunity to mobilise CBOs and FBOs has been recognised.

### Media

The *Ethiopian* media was regarded as having medium levels of interest and being supportive of the issue at a national level. Variable levels of interest amongst District media, and opportunities to engage this sector have been recorded.

Although regarded as highly influential, the media in *India* were expected to have low levels of interest in the issue. Opportunities to mobilise them further were recognised.

*Nepalese* media were believed to have medium–high interest in the issue, and to be highly supportive of the issue, particularly from the perspective of sensitization and awareness.

Whilst *South African* media had not yet been mobilised regarding the issue, medium levels of interest, and high levels of influence were expected, particularly in terms of placing the issue higher on the policy and implementation agenda.

The *Ugandan* national media was believed to have a medium interest in the issue, and was identified as being supportive, with high levels of influence, and impact on the actor. Lower levels of interest were recorded amongst District level media, and opportunities to mobilise media at this level were recorded.

### Academics

Although academics and researchers in *Ethiopia* (such as high level officials in universities) were regarded as being supportive of the issue, the level of interest was believed to be low-medium given that mental health programmes were not prioritised. However, the potential influence on the issue was noted as high, as mental health issues could become mainstreamed into education and training at the universities.

In *India*, universities were regarded as having high levels of interest in order to enhance the academic pool of resources, however, other research institutes were believed to be less interested in the issue given their objectives, priorities and links with government.

*Nepal* regarded universities and other research communities as having high levels of interest, and support for the issue due to the potential impact that the scaling-up of mental health services has on their development agenda.

*South Africa* regarded universities and research communities as having medium levels of interest, with some academics having specific interests in mental health, whilst others, more general interests in public health. The potential influence of universities and research communities regarding the issue was recognised, as more research was being published about scaling-up mental health care, which was believed to have a greater impact.

*Uganda* believed universities to be supportive, with a medium interest in the issue. Lower interest was anticipated amongst other research communities, and the opportunity to mobilise them has been noted.

### Cross-country force field analysis map: summarising stakeholder engagement opportunities

Content analysis of the stakeholder analysis tables identified a range of opportunities for increasing the evidence-based scale-up of mental health care across countries. However, not all of these may succeed given varying degree of support and power of stakeholders.

Hence, a cross-country force field analysis (Table 1) indicating the extent of stakeholder support (across) and their perceived power (down) has been applied. The block on the top right indicates those stakeholders that are the most influential and supportive of scaling-up mental health care, whilst the bottom left block indicates the least influential, least supportive stakeholders.

**Table 1:** Cross-country stakeholder forcefield analysis map

Power	Opposition	Not mobilised	Mostly supportive	Supportive
High		Parliament (IN, UG)	MH Specialists, PHC Workers, CHW (SA)	WHO, MoH (ET, NP, UG)
		PHC Workers (UG)		Parliament (ET)
		Volunteer Workers (IN)		DFID UK (ET, NP, UG)
		Persons with mental illness, Families (ET, IN)		DFID local (ET)
		Service User groups (IN)		Other donors (NP)
		CBOs (ET)		MH Specialists (UG)
		I-Media (SA)		N-Media (NP, UG)
		R-Media (ET, SA)		State/District Media (NP)
		N-Media (IN, SA)		Universities (ET)
		State/District Media (ET, IN, SA)		
Medium–high				Non-Health Ministries (ET)
				MH Specialists (ET)
				Service User groups (NP)
				INGOs, NGOs, CBOs (NP)
				FBOs (ET)
				N-Media (ET)
Medium		Non-Health Ministries (NP, UG)		WHO, MoH (IN, SA)
		CHW (UG)		Non-Health Ministries, Parliament (SA)
		Persons with mental illness (UG)		DFID UK (IN, SA)
		CBOs, FBOs (IN)		DFID local (IN, NP, SA)
		I-Media (IN)		Other donors (ET, SA)
		State/District Media (UG)		CHW (ET)
		Universities (IN, SA)		Service User groups (UG)
				INGOs (UG)
				NGOs (IN, SA)
				CBOs, FBOs (SA)
				Universities (UG)
Low–medium		MH Specialists, PHC Workers, CHW (IN)		MH Specialists (NP)
				PHC Workers (ET)
				Persons with mental illness, Families (NP)
Low		Non-Health Ministries (IN)	FBOs (NP)	PHC in MoH (NP)
		DFID local (UG)		PHC Workers, CHW, Volunteer Workers (NP)
		Other donors (IN, UG)		Service User groups (ET)
		Persons with mental illness, Families (SA)		INGOs (ET)
		Families of persons with mental illness (UG)		I-Media (NP)
		INGOs (IN)		Universities, Research Institutes (NP)
		CBOs, FBOs (UG), Research Institutes (UG)		

Perceived power to influence the scaling up of mental health care (down) by position relating to the scale up of mental health care (across).

*ET* Ethiopia, *IN* India, *NP* Nepal, *SA* South Africa, *UG* Uganda.

In terms of priority setting in the context of limited resources, it can be deduced that success amongst the mobilised will be most likely amongst highly influential (powerful) stakeholders, who are most supportive. Should further capacity exist, medium-highly influential stakeholders that are supportive can be targeted next, followed by the supportive medium influential stakeholders, or the highly influential non-mobilised stakeholders. Although it was early in the programme and many stakeholders were not mobilised at the time of this research, it is noteworthy that none of the key informants recorded any opposition or resistance to scaling-up mental health care, indicating that there is no direct opposition to the issue from a wide range of stakeholders.

In *Ethiopia*, the policy engagement opportunities with the WHO regional office, Federal Ministry of Health and Parliament could be prioritised. In the case of other stakeholders, the donor sector (DFID UK and local offices) and universities could also be prioritised.

In the case of *India*, policy engagement opportunities could be prioritised with the WHO regional office; Ministry of Health and Family Welfare and AIDS control organisation (national level); Department of Medical Education, state mental health authority and state AIDS control society in Madhya Pradesh (district level). Other opportunities identified that could be prioritised included the donor sector (DFID UK and local offices) and national NGOs.

*Nepal* could prioritise its policy engagement activities with the WHO and the Ministry of Health, including PHC revitalisation department. Other engagements could be prioritised with the donor sector (DFID UK and other donors), national and district media, service user groups, international and national NGOs and CBOs.

Policy engagement priorities in *South Africa* could be with the local WHO, Department of Health (national), non-health sector departments (Department of Social Development) and Parliament. Other stakeholders that could be prioritised by South Africa include health practitioners (mental health specialists, PHC workers and CHWs), donors (DFID UK and other donors) and civil society (NGOs, CBOs and FBOs).

*Uganda’s* policy engagement priorities could be with the local WHO and Ministry of Health. Other stakeholder engagement priorities could be with donors, mental health specialists, national media (high power); followed by service user groups, international NGOs and universities (medium power).

## Discussion

### Stakeholder analysis: use in identifying stakeholders and opportunities for research uptake

The stakeholder analysis method, with the participation of diverse country and cross-country partners, provided a useful means of identifying a range of specific stakeholders within countries. From a policy perspective, most countries report high levels of support and interest from the WHO with the exception of Nepal, which reports a medium level of interest from WHO country offices, despite being supportive. Ministries of Health, as active partners involved since the inception of PRIME, are reported to have high levels of support and interest in most countries, with the exception of India and South Africa which report medium levels of support and interest due to other programmes having priority. This is in contrast with the low policy attention mental health has traditionally received [10–13]. Opportunities exist for the Indian and South African teams to align PRIME with other Ministry of Health programmes, such as with HIV/AIDS in South Africa [22]. The identification of stakeholders in countries has been particularly useful for identifying other non-health public policy actors that may have an interest in scaling up mental health care, and in supporting the implementation of PRIME, pointing to the importance of intersectoral collaboration and adopting a Health in All Policies (HiAP) approach [23]. Specifically, the Ministry of Social and Labour Affairs, the Ministry of Women and Children Affairs and the Ministry of Youth and Sport in Ethiopia; the National AIDS Control Organisation in India; the Ministry of Women, Children and Social Welfare, and the Ministry of Peace and Reconstruction in Nepal; the Department of Social Development in South Africa have been identified, and could be explored for strengthening collaborations.

Although specialist mental health practitioners’ (psychiatrists and psychologists) across PRIME countries were commonly believed to have high levels of support, general health practitioners (primary health care workers, community and voluntary health workers) were believed to have low-medium levels of support and interest due to being overburdened, which has also been widely documented [15, 16, 24, 25]. This has important implications for integrating mental health into public health facilities, and creates opportunities for sensitising human resources for mental health, strategies of which are also well documented [20, 24].

With regards to persons affected by mental illness (including their families and carers), countries record mixed levels of interest and support, with Ethiopia and Nepal recording particularly high levels of interest and support from service user groups. Where support is low, service users should be actively encouraged to participate in mental health policy and service reform. This will not only improve the integration of mental health into primary care, but also support their recovery process [26].

Amongst civil society organisations, high levels of support have been reported amongst NGOs and CBOs. Although their role has been acknowledged [27], greater efforts to engage this sector will contribute towards a more integrated health system. Where mobilised (Ethiopia, Nepal and SA), FBOs were regarded as mostly supportive, with medium to high levels of power. This is important given the fact that traditional healers or religious advisors are often the first to see persons living with mental illness. The role and importance of engaging with this group, and of finding ways of blending traditional practices with modern medicine has been widely recognised [28–30].

Where mobilised, high levels of support and power have been identified amongst the media in Nepal and Uganda. The role and power of the media to influence public perceptions regarding mental health has been documented [31]. Opportunities to engage with media should be maximised.

Universities and research institutes are generally believed to be supportive of the issue, with varying levels of power in countries. Continued engagement with universities and research communities will help to facilitate increased support for scaling-up mental health care from those working in public health, and other related faculties and disciplines such as social sciences, economics, political science and religious studies.

At a country level, stakeholder analysis proved a useful technique to identify specific stakeholders, their interests, positions and power, and accordingly, opportunities for increased stakeholder engagement [1–4]. These strategies should be assessed for feasibility by consultation with PRIME country teams, and if feasible, incorporated into PRIME’s research uptake strategy.

PRIME’s cross-country stakeholder analysis reveals generally high levels of support amongst WHO and Ministries of Health. This may be explained by the fact that these stakeholders are partners in PRIME. DFID is also identified by most countries as having high levels of support based on their funding and acceptance of the research programme proposal. Where mobilised, health practitioners, persons affected by mental illness, civil society, media and academics also tend to be generally supportive. Despite the fact that mental health receives little policy attention [10], no stakeholder opposition to the issue has been recorded. This may be due to the fact that some stakeholders were not mobilised at the time, and where mobilised, it may have been recorded as such for diplomatic reasons, and in order not to jeopardise engagement opportunities in the future life of the programme.

Despite the inclusion of a wide range of stakeholder groups in the above analysis, it is apparent that some groups, which may have the potential of introducing policy windows or barriers, may have been omitted. In conducting a stakeholder analysis to support moves for health insurance reform in South Africa and Tanzania, Gilson and colleagues [4] identify a number of stakeholder groups that are potentially highly influential that have not been considered in PRIME. These include political parties, the private healthcare system and business, some of which may explain the lack of any recorded opposition to the scaling up of mental health services. These groups of stakeholders, and their interest, position and influence in relation to the issue should be incorporated into the framework, and considered for the next stakeholder analysis planned for the programme.

### Force field analysis: Identifying stakeholder engagement opportunities

Force field analysis has been a useful tool to explore and understand stakeholder motivations and to prioritise stakeholder engagements. However, the prioritisation of these potential engagement opportunities should not be depended upon entirely in light of the fact that a large number of stakeholders were not mobilised at the time of data collection.

True to the findings of Gilson et al. [4], force field analysis has been helpful to summarise stakeholder analyses and provide a comprehensive picture of the balance of support and power around a policy issue at a particular time, and for developing a ‘political management strategy’. More specifically, applying force field analysis to PRIME’s stakeholder analysis has been an invaluable technique for prioritising the stakeholders to focus on.

Cross-country stakeholder analyses of PRIME have demonstrated the usefulness of this approach to illuminate the host of opportunities available to narrow the gap between research, and its translation into knowledge, including policy and practice. However, it is acknowledged that not all strategies may be feasible to implement, and are dependent on the programme’s capacity and resources. In such cases, a force field analysis is an invaluable tool for prioritising the stakeholder engagement strategies likely to be most successful.

### Limitations

The study has several limitations. The *first* relates to the method of data collection. In a paper on using stakeholder analysis to support moves toward universal coverage in two countries, Gilson et al. [4] indicate that stakeholder analysis can be conducted by gathering data through document review, media analysis and in-depth interviews, or by brainstorming with knowledgeable participants. In the case of PRIME, stakeholder analysis was conducted by gathering information from knowledgeable participants. Knowledgeable participants were local PRIME teams within countries, including the Principal Investigators (psychiatrists or psychologists), project coordinators and district coordinators (Masters qualified) who have had past or current experience of regularly engaging with stakeholders. Whilst brainstorming happened face-to-face within countries, the lack of face-to-face engagement by the lead researcher with knowledgeable participants across countries may have resulted in reduced data quality. The *second* relates to the choice of key informants. Although spanning a range of research institutions, some may regard the key informants as ‘insiders’ [20] since they are partners in the PRIME consortium, and hence may bias their responses. With the necessary resources, one way to address this concern is by conducting actual interviews or focus group discussions with external stakeholders, or by seeking opportunities to do so within PRIME’s research methodology by, for instance, mapping stakeholder inputs from the Theory of Change workshops conducted with stakeholders. *Thirdly*, the manner in which the data were recorded was inconsistent, with some countries providing stakeholder characteristics at a category level (e.g. same characteristics for all policy makers), whilst others provided characteristics for specific stakeholders (e.g. Ministry of Health). Another data recording inconsistency related to the amount of additional qualitative information that was yielded from the analyses, with most informants providing explanations for stakeholder positions. A more detailed guideline and survey instrument for completing the stakeholder table is likely to yield higher quality data in future.

Despite the limitations identified, stakeholder analysis has been significant in providing a systematic means of identifying, and documenting the position of stakeholders in relation to the scaling-up of mental health care into health systems in five LMICs. The findings regarding its utility were largely consistent with most authors in the field, and force field analysis was a useful technique to prioritise stakeholders for the purposes of strategic research uptake management.

### Future research

Given that stakeholder analysis is an indication of stakeholder positions and power at a single point in time, PRIME intends to repeat this process as part of its research uptake strategy during the scaling-up phase of the programme in order to measure changes over the life of the programme, and in so doing, shed some light on the extent to which research may have translated into policy and practice. Citing the success of integrating HIV research into policy and practice, Collins et al. appropriately call for a global, coordinated uptake of data driven findings [12]. The formulation of a cross-country research uptake strategy was a useful means of collecting data for stakeholder analysis, and coordinating the uptake of evidence-based research amongst PRIME stakeholders at a programme level. Opportunities exist for conducting further stakeholder analysis across global mental health research programmes, and in turn, for including this into research uptake and communication strategies to scale up and integrate mental health into primary health care for the improvement of health systems.

## Conclusion

Stakeholder analysis of PRIME has identified policy makers (WHO, Ministries of Health, other non-health sector Ministries and Parliament), donors (DFID UK, DFID country offices and other donor agencies), mental health specialists, the media (national and district) and universities as the most powerful, and most supportive actors for scaling up mental health care in the respective PRIME countries. Stakeholder analysis has been valuable in identifying, characterising and mobilising support for the proposed action. Force field analysis has not only been a useful means of summarising stakeholder power and positions regarding the scaling up of mental health services, but has also demonstrated its relevance as a tool to prioritise engagement activities amongst the already mobilised stakeholders. The prioritisation of stakeholders is of particular importance in maximising the efficiency of limited programme resources, strategically supporting the research uptake management process. We hope that the insights gained from stakeholder analysis regarding the scale up of mental health care in low and middle-income countries is likely to result in more targeted strategies for stimulating the demand for research amongst knowledge users. In theory, this should result in improved translation of evidence-based research into policy and practice, and in turn, better mental health outcomes.

## Additional files

Additional file 1:
**Table S1.** Policymakers: Cross-country stakeholder characteristics regarding the scale-up of mental health care. Country Key: ET – Ethiopia; IN – India; NP – Nepal; SA – South Africa; UG – Uganda (ranked High-Low; Supportive-Opposed or NonMob – Not yet mobilised). Additional file 2:
**Table S2.** Donors: Cross-country stakeholder characteristics regarding the scale-up of mental health care. Country Key: ET – Ethiopia; IN – India; NP – Nepal; SA – South Africa; UG – Uganda (ranked High-Low; Supportive-Opposed or NonMob – Not yet mobilised). Additional file 3:
**Table S3.** Health Practitioners: Cross-country stakeholder characteristics regarding the scale-up of mental health care. Country Key: ET – Ethiopia; IN – India; NP – Nepal; SA – South Africa; UG – Uganda (ranked High-Low; Supportive-Opposed or NonMob – Not yet mobilised). Additional file 4:
**Table S4.** Persons affected by Mental Illness: Cross-country stakeholder characteristics regarding the scale-up of mental health care. Country Key: ET – Ethiopia; IN – India; NP – Nepal; SA – South Africa; UG – Uganda (ranked High-Low; Supportive-Opposed or NonMob – Not yet mobilised). Additional file 5:
**Table S5.** Civil Society: Cross-country stakeholder characteristics regarding the scale-up of mental health care. Country Key: ET – Ethiopia; IN – India; NP – Nepal; SA – South Africa; UG – Uganda (ranked High-Low; Supportive-Opposed or NonMob – Not yet mobilised). Additional file 6:
**Table S6.** Media: Cross-country stakeholder characteristics regarding the scale-up of mental health care. Country Key: ET – Ethiopia; IN – India; NP – Nepal; SA – South Africa; UG – Uganda (ranked High-Low; Supportive-Opposed or NonMob – Not yet mobilised). Additional file 7:
**Table S7.** Academics: Cross-country stakeholder characteristics regarding the scale-up of mental health care. Country Key: ET – Ethiopia; IN – India; NP – Nepal; SA – South Africa; UG – Uganda (ranked High-Low; Supportive-Opposed or NonMob – Not yet mobilised).

## Abbreviations

ASHAs: Accredited Social Health Assistants (India)
CBOs: Community-Based Organisations
CHWs: Community-Health Workers
ET: Ethiopia
FBOs: Faith-Based Organisations
FCHVs: Female Community Health Volunteers (Nepal)
HiAP: Health in All Policies
IN: India
NGOs: Non-Governmental Organisations
NP: Nepal
PHC: primary health care
PRIME: PRogramme for Improving Mental health carE
SHA: stakeholder analysis
SA: South Africa
UG: Uganda
