# Supplementary material for: Stakeholder analysis of the Programme for Improving Mental health carE (PRIME): baseline findings
Source: Int J Ment Health Syst. 2015 Jul 8;9:27. doi: 10.1186/s13033-015-0020-z (PMC4493963; doi:10.1186/s13033-015-0020-z)
Supplement: Additional file 1: — Table S1. Policymakers: Cross-country stakeholder characteristics regarding the scale-up of mental health care. Country Key: ET – Ethiopia; IN – India; NP – Nepal; SA – South Africa; UG – Uganda (ranked High-Low; Supportive-Opposed or NonMob – Not yet mobilised). [file 13033_2015_20_MOESM1_ESM.docx]

| **TABLE S1: POLICY MAKERS - CROSS-COUNTRY STAKEHOLDER CHARACTERISTICS REGARDING THE SCALE-UP OF MENTAL HEALTH CARE** | | | | | |
| --- | --- | --- | --- | --- | --- |
| **Stakeholder** | **Involvement in the Issue** | **Interest in the Issue (low, medium, high)** | **Influence/power (low, medium, high)** | **Position**  **(supportive, opposed, non-mobilised)** | **Impact of Issue on Actor (low, medium, high)** |
| World Health Organisation (WHO) | WHO Department of Mental Health and Substance Abuse is a cross-country partner in PRIME, and perform a function in terms of formulating global mental health policy. | ET – High  IN - High  UG - High  NP - Med  SA – Med | ET – High  NP – High  UG - High  IN – Med  SA – Med | ET – Support  IN – Support  NP – Support  SA - Support  UG - Support | ET – Med/High  IN – Med  SA – Med  UG – Med  NP – Low/Med |
| Ministries of Health (MoH) | Country Ministries of Health are partners in PRIME and are primarily responsible for policy formulation at a national level, and policy implementation, monitoring and supervision at a state/district levels. | ET – High  UG - High  IN – Med  SA - Med  NP – Low | ET – High  NP – High  UG – High  SA – Med  IN – Low/Med | ET- Support  IN – Support  NP – Support  SA - Support  UG - Support | ET – High  UG - High  NP – Med/High  SA – Med  IN – Low/Med |
| Primary Health Care (PHC) Agencies within MoH | Responsible for PHC Policy Implementation within the MoH. | NP - High | NP - Low | NP - Support | NP - High |
| Non-health sector Ministries (e.g. social, economic, development) | Other non-health sector government Ministries, such as social, economic or development, may be involved in policies or programmes that address mental health. | NP – High  ET – Medium  SA – Med  IN – Low  UG - Low | ET – Med/High  NP – Med  SA – Med  UG – Med  IN – Low | ET – Support  SA – Support  IN – NonMob  NP – NonMob  UG - NonMob | ET – Med  SA – Med  NP – Low/Med  IN – Low  UG - Low |
| Parliament / other democratic institutions | Parliament and other institutions of democracy, through executive oversight, may perform the function of influencing policies relating to mental health. | ET – Med  SA – Med  IN – Low  UG - Low | ET – High  IN – High  UG - High  SA – Med | ET – Support  SA – Support  IN – NonMob  NP - NonMob  UG - NonMob | ET – High  IN – High  SA – Med  UG - Low |

Country Key: ET – Ethiopia; IN – India; NP – Nepal; SA – South Africa; UG – Uganda (ranked High-Low; Supportive-Opposed)
