# Supplementary material for: Stakeholder analysis of the Programme for Improving Mental health carE (PRIME): baseline findings
Source: Int J Ment Health Syst. 2015 Jul 8;9:27. doi: 10.1186/s13033-015-0020-z (PMC4493963; doi:10.1186/s13033-015-0020-z)
Supplement: Additional file 2: — Table S2. Donors: Cross-country stakeholder characteristics regarding the scale-up of mental health care. Country Key: ET – Ethiopia; IN – India; NP – Nepal; SA – South Africa; UG – Uganda (ranked High-Low; Supportive-Opposed or NonMob – Not yet mobilised). [file 13033_2015_20_MOESM2_ESM.docx]

| **TABLE S2: DONORS - CROSS-COUNTRY STAKEHOLDER CHARACTERISTICS REGARDING THE SCALE-UP OF MENTAL HEALTH CARE** | | | | | |
| --- | --- | --- | --- | --- | --- |
| **Stakeholder** | **Involvement in the Issue** | **Interest in the Issue (low, medium, high)** | **Influence/power (low, medium, high)** | **Position**  **(supportive, opposed, non-mobilised)** | **Impact of Issue on Actor (low, medium, high)** |
| Department for International Development (DFID) | DFID is involved as the donor of the research programme, and performs a role of monitoring and overseeing the implementation of the programme. | ET – High  NP – High  UG - High  IN – Med  SA – Med | ET – High  NP – High  UG – High  IN – Med  SA – Med | ET- Support  IN – Support  NP – Support  SA – Support  UG - Support | ET – High  IN – High  SA – High  NP – Med/High  UG - Med |
| DFID Regional/ Country Offices | Due the fact that DFID funds the programme, regional or country offices may be interested in the work of the programme. | ET – High  IN – Med  NP – Med  SA – Med  UG - Low | ET – High  IN – Med  NP – Med  SA – Med  UG - Low | ET- Support  IN – Support  NP – Support  SA – Support  UG - NonMob | ET – High  IN – High  SA – High  NP – Med/High  UG - Low |
| Other Donors / Development Agencies | Other donors interested in funding global mental health, and its integration into primary health care may have an interest in the work of the programme. | ET -Med/High  NP – Med  SA – Med  UG – Med  IN – Low | NP – High  ET - Med  SA – Med  IN – Low  UG - Low | ET- Support  NP – Support  SA – Support  IN – NonMob  UG - NonMob | SA – High  ET – Medium  NP – Low/Med  IN – Low  UG - Low |

Country Key: ET – Ethiopia; IN – India; NP – Nepal; SA – South Africa; UG – Uganda (ranked High-Low; Supportive-Opposed)
