# Supplementary material for: Stakeholder analysis of the Programme for Improving Mental health carE (PRIME): baseline findings
Source: Int J Ment Health Syst. 2015 Jul 8;9:27. doi: 10.1186/s13033-015-0020-z (PMC4493963; doi:10.1186/s13033-015-0020-z)
Supplement: Additional file 3: — Table S3. Health Practitioners: Cross-country stakeholder characteristics regarding the scale-up of mental health care. Country Key: ET – Ethiopia; IN – India; NP – Nepal; SA – South Africa; UG – Uganda (ranked High-Low; Supportive-Opposed or NonMob – Not yet mobilised). [file 13033_2015_20_MOESM3_ESM.docx]

| **TABLE S3: HEALTH PRACTITIONERS - CROSS-COUNTRY STAKEHOLDER CHARACTERISTICS REGARDING THE SCALE-UP OF MENTAL HEALTH CARE** | | | | | |
| --- | --- | --- | --- | --- | --- |
| **Stakeholder** | **Involvement in the Issue** | **Interest in the Issue (low, medium, high)** | **Influence/power (low, medium, high)** | **Position**  **(supportive, opposed, non-mobilised)** | **Impact of Issue on Actor (low, medium, high)** |
| Mental health specialists (psychiatrists & psychologists) | Mental health specialists are involved in providing specialised care for persons affected by mental illness through supervision and training of Primary Health Care workers. | ET – High  UG - High  IN – Med  NP – Med  SA – Med | SA – High  UG - High  ET -Med/High  IN – Low/Med  NP – Low/Med | ET- Support  NP – Support  UG – Support  SA – Mostly Support  IN – NonMob | IN – High  SA – High  UG – High  NP – Med/High  ET – Med |
| Primary Health Care (PHC) workers (doctors, nurses & health workers) | PHC workers are responsible for delivering general primary health care service to patients, and are needed to provide mental health care, and appropriately refer serious mental illnesses through mhGAP training. | NP – Med/High  ET – Med  IN – Med  SA – Med  UG - Low | SA – High  UG - High  ET – Low/Med  IN – Low/Med  NP – Low | ET- Support  NP – Support  SA – Mostly Support  IN – NonMob  UG - NonMob | ET – High  IN – High  NP – High  SA – High  UG - Med |
| Community Health Workers (CHWs) | Health workers residing in communities which perform the roles of raising awareness and identifying mental illness, and providing a basic level of psychosocial care. | NP – Med/High  ET – Med  IN – Med  SA – Med  UG - Low | SA – High  ET – Med  UG - Med  IN – Low/Med  NP – Low | ET- Support  NP – Support  SA – Mostly Support  IN – NonMob  UG - NonMob | IN – High  NP – High  SA – High  ET – Med/High  UG - Med |
| Voluntary Health Workers (e.g. ASHA in India, FCHV in Nepal) | Responsible for psychosocial education in communities. | NP – Med/High  IN – Med | IN – High  NP – Low | NP – Support  IN – NonMob | IN – High  NP – High |

Country Key: ET – Ethiopia; IN – India; NP – Nepal; SA – South Africa; UG – Uganda (ranked High-Low; Supportive-Opposed)
