# Supplementary material for: Stakeholder analysis of the Programme for Improving Mental health carE (PRIME): baseline findings
Source: Int J Ment Health Syst. 2015 Jul 8;9:27. doi: 10.1186/s13033-015-0020-z (PMC4493963; doi:10.1186/s13033-015-0020-z)
Supplement: Additional file 4: — Table S4. Persons affected by Mental Illness: Cross-country stakeholder characteristics regarding the scale-up of mental health care. Country Key: ET – Ethiopia; IN – India; NP – Nepal; SA – South Africa; UG – Uganda (ranked High-Low; Supportive-Opposed or NonMob – Not yet mobilised). [file 13033_2015_20_MOESM4_ESM.docx]

| **TABLE S4: PERSONS AFFECTED BY MENTAL ILLNESS - CROSS-COUNTRY STAKEHOLDER CHARACTERISTICS REGARDING THE SCALE-UP OF MENTAL HEALTH CARE** | | | | | |
| --- | --- | --- | --- | --- | --- |
| **Stakeholder** | **Involvement in the Issue** | **Interest in the Issue (low, medium, high)** | **Influence/power (low, medium, high)** | **Position**  **(supportive, opposed, non-mobilised)** | **Impact of Issue on Actor (low, medium, high)** |
| Service user groups | Service user groups act as a voice for mental health service users, and are involved in advocacy, sensitisation and raising awareness of mental health issues. | ET – High  NP – High  UG – High  IN – Med | IN – High  NP – Med/High  UG – Med  ET – Low | ET- Support  NP – Support  UG – Support  IN – NonMob | IN – High  NP – Med/High  UG – Med  ET – Low/Med |
| Persons with psychosocial disabilities (including MNS mental, neurological and substance use disorders) | Persons with psychosocial disabilities are involved in that they are the direct target beneficiaries of the mental health service. | ET – High  NP – High  IN – Med  SA – Med  UG - Med | ET – High  IN – High  UG - Med  NP – Low/Med  SA – Low | NP – Support  ET – NonMob  IN – NonMob  SA – NonMob  UG - NonMob | ET – High  IN – High  SA – High  NP – Med/High  UG - Low |
| Family/carers of persons with psychosocial disabilities | Families and carers of persons with psychosocial disabilities are involved in helping persons seek mental health care and hence, reducing their own burden of care. Families may also be a barrier to recover due to stigma and discrimination. | ET – High  NP – High  SA – High  IN – Med  UG - Low | ET – High  IN – High  NP – Low/Med  SA – Low  UG - Low | NP – Support  ET – NonMob  IN – NonMob  SA – NonMob  UG - NonMob | ET – High  IN – High  SA – High  NP – Med/High  UG - Low |

Country Key: ET – Ethiopia; IN – India; NP – Nepal; SA – South Africa; UG – Uganda (ranked High-Low; Supportive-Opposed)
