# Supplementary material for: Stakeholder analysis of the Programme for Improving Mental health carE (PRIME): baseline findings
Source: Int J Ment Health Syst. 2015 Jul 8;9:27. doi: 10.1186/s13033-015-0020-z (PMC4493963; doi:10.1186/s13033-015-0020-z)
Supplement: Additional file 5: — Table S5. Civil Society: Cross-country stakeholder characteristics regarding the scale-up of mental health care. Country Key: ET – Ethiopia; IN – India; NP – Nepal; SA – South Africa; UG – Uganda (ranked High-Low; Supportive-Opposed or NonMob – Not yet mobilised). [file 13033_2015_20_MOESM5_ESM.docx]

| **TABLE S5: CIVIL SOCIETY - CROSS-COUNTRY STAKEHOLDER CHARACTERISTICS REGARDING THE SCALE-UP OF MENTAL HEALTH CARE** | | | | | |
| --- | --- | --- | --- | --- | --- |
| **Stakeholder** | **Involvement in the Issue** | **Interest in the Issue (low, medium, high)** | **Influence/power (low, medium, high)** | **Position**  **(supportive, opposed, non-mobilised)** | **Impact of Issue on Actor (low, medium, high)** |
| International Non-Governmental Organisations (NGOs) | International NGOs advocating for public mental health, or providing psychosocial support services have a role to play in the health system of countries. | ET – Medium  IN – Low  NP – Med  SA – High  UG - Med | ET – Low  IN – Low  NP – Med/High  UG - Med | ET- Support  IN – NonMob  NP – Support  UG - Support | ET – Low  IN – Med  NP – Med/High  UG - Med |
| National NGOs | National NGOs advocating for public mental health, or providing psychosocial support services have a role to play in the health system of the country. | IN – Med/High  NP – High  SA - High | IN – Med  NP – Med/High  SA - Med | IN – Support  NP – Support  SA - Support | IN – Med  NP – Med/High  SA - High |
| Community Based Organisations (CBOs) | CBOs are involved in community-level advocacy and mobilisation and may be able to perform a role in referring persons with mental illness to the mental health service. | ET – Medium/High  IN – Med  NP – High  SA – High  UG - Low | ET – High  IN – Med  NP – Med/High  SA – Med  UG - Low | ET – NonMob  IN – NonMob  NP – Support  SA – Support  UG - NonMob | ET – Medium  IN – High  NP – Med/High  SA – High  UG - Low |
| Faith Based Organisations, including traditional healers (FBOs) | FBOs are involved in providing support for persons seeking faith-based help, and may be able to perform a role in referring persons with mental illness to the mental health service. | ET – Medium  IN - Medium  NP – Low/Med  SA – High  UG - Low | ET – Med/High  IN – Med  NP – Low  SA – Med  UG - Low | ET- Support  IN – NonMob  NP – Less Support  SA – Support  UG - NonMob | ET – Med/High  IN – Low  NP – Low/Med  SA – High  UG - Low |

Country Key: ET – Ethiopia; IN – India; NP – Nepal; SA – South Africa; UG – Uganda (ranked High-Low; Supportive-Opposed)

| **TABLE S6: MEDIA - CROSS-COUNTRY STAKEHOLDER CHARACTERISTICS REGARDING THE SCALE-UP OF MENTAL HEALTH CARE** | | | | | |
| --- | --- | --- | --- | --- | --- |
| **Stakeholder** | **Involvement in the Issue** | **Interest in the Issue (low, medium, high)** | **Influence/power (low, medium, high)** | **Position**  **(supportive, opposed, non-mobilised)** | **Impact of Issue on Actor (low, medium, high)** |
| International / Global | International media performs a role in terms of influencing public perceptions relating to mental illnesses, and mental health care | IN – Low  SA - Med | IN – Med  SA – High  NP - Low | NP - Support  IN – NonMob  SA - NonMob | IN – Low  SA - Low |
| Regional | Regional media performs a role in terms of influencing public perceptions relating to mental illnesses, and mental health care | ET – Low/Med  SA - Med | ET – High  SA – High  NP - Low | NP - Support  ET – NonMob  SA - NonMob | ET – Unknown  SA - Low |
| National | National media performs a role in terms of influencing public perceptions relating to mental illnesses, and mental health care | ET – Low/Med  IN – Low  NP – Med/High  SA – Med  UG - Med | IN – High  NP – High  SA – High  UG – High  ET – Medium/High | ET- Support  NP – Support  UG – Support  IN – NonMob  SA – NonMob | ET – Medium/High  IN – Low  SA – Low  UG - High |
| Provincial/State | Provincial/State media performs a role in terms of influencing public perceptions relating to mental illnesses, and mental health care | ET – Low/High  IN – Low  SA - Med | ET – High  IN – High  SA - High | ET – NonMob  IN – NonMob  SA - NonMob | ET – Medium  IN – Low  SA - Low |
| Local (District) | Local media performs a role in terms of influencing public perceptions relating to mental illnesses, and mental health care | IN – Low  NP – Med/High  SA – Med  UG - Low | IN – High  NP – High  SA – High  UG - Med | NP – Supportive  IN – NonMob  SA – NonMob  UG - NonMob | IN - Low  SA – Low  UG - Med |

Country Key: ET – Ethiopia; IN – India; NP – Nepal; SA – South Africa; UG – Uganda (ranked High-Low; Supportive-Opposed)

| **TABLE S7: ACADEMICS - CROSS-COUNTRY STAKEHOLDER CHARACTERISTICS REGARDING THE SCALE-UP OF MENTAL HEALTH CARE** | | | | | |
| --- | --- | --- | --- | --- | --- |
| **Stakeholder** | **Involvement in the Issue** | **Interest in the Issue (low, medium, high)** | **Influence/power (low, medium, high)** | **Position**  **(supportive, opposed, non-mobilised)** | **Impact of Issue on Actor (low, medium, high)** |
| Universities | Universities are involved in teaching, producing and disseminating research evidence, and can perform a role in terms of using PRIME as a case study and integrating public mental health into their curricula | IN – High  NP – High  SA – Med  UG – Med  ET - Low/Med | ET – High  IN – Med  SA – Med  UG – Med  NP – Low | ET – Support  NP – Support  UG – Support  IN – NonMob | ET – High  SA – High  UG – Med  IN – Med |
| Research Institutions | Research Institutions are involved in producing and disseminating research evidence and can perform a role in integrating mental health into their research agenda | NP – High  SA – Med  UG – Low  IN – Low | IN – Med  SA – Med  UG – Low  NP – Low | NP – Supportive  IN – NonMob  UG - NonMob | SA – High  NP – Med/High  IN – Low  UG - Low |

Country Key: ET – Ethiopia; IN – India; NP – Nepal; SA – South Africa; UG – Uganda (ranked High-Low; Supportive-Opposed
