# Supplementary material for: Stakeholder analysis of the Programme for Improving Mental health carE (PRIME): baseline findings
Source: Int J Ment Health Syst. 2015 Jul 8;9:27. doi: 10.1186/s13033-015-0020-z (PMC4493963; doi:10.1186/s13033-015-0020-z)
Supplement: Additional file 6: — Table S6. Media: Cross-country stakeholder characteristics regarding the scale-up of mental health care. Country Key: ET – Ethiopia; IN – India; NP – Nepal; SA – South Africa; UG – Uganda (ranked High-Low; Supportive-Opposed or NonMob – Not yet mobilised). [file 13033_2015_20_MOESM6_ESM.docx]

| **TABLE S6: MEDIA - CROSS-COUNTRY STAKEHOLDER CHARACTERISTICS REGARDING THE SCALE-UP OF MENTAL HEALTH CARE** | | | | | |
| --- | --- | --- | --- | --- | --- |
| **Stakeholder** | **Involvement in the Issue** | **Interest in the Issue (low, medium, high)** | **Influence/power (low, medium, high)** | **Position**  **(supportive, opposed, non-mobilised)** | **Impact of Issue on Actor (low, medium, high)** |
| International / Global | International media performs a role in terms of influencing public perceptions relating to mental illnesses, and mental health care | IN – Low  SA - Med | IN – Med  SA – High  NP - Low | NP - Support  IN – NonMob  SA - NonMob | IN – Low  SA - Low |
| Regional | Regional media performs a role in terms of influencing public perceptions relating to mental illnesses, and mental health care | ET – Low/Med  SA - Med | ET – High  SA – High  NP - Low | NP - Support  ET – NonMob  SA - NonMob | ET – Unknown  SA - Low |
| National | National media performs a role in terms of influencing public perceptions relating to mental illnesses, and mental health care | ET – Low/Med  IN – Low  NP – Med/High  SA – Med  UG - Med | IN – High  NP – High  SA – High  UG – High  ET – Medium/High | ET- Support  NP – Support  UG – Support  IN – NonMob  SA – NonMob | ET – Medium/High  IN – Low  SA – Low  UG - High |
| Provincial/State | Provincial/State media performs a role in terms of influencing public perceptions relating to mental illnesses, and mental health care | ET – Low/High  IN – Low  SA - Med | ET – High  IN – High  SA - High | ET – NonMob  IN – NonMob  SA - NonMob | ET – Medium  IN – Low  SA - Low |
| Local (District) | Local media performs a role in terms of influencing public perceptions relating to mental illnesses, and mental health care | IN – Low  NP – Med/High  SA – Med  UG - Low | IN – High  NP – High  SA – High  UG - Med | NP – Supportive  IN – NonMob  SA – NonMob  UG - NonMob | IN - Low  SA – Low  UG - Med |

Country Key: ET – Ethiopia; IN – India; NP – Nepal; SA – South Africa; UG – Uganda (ranked High-Low; Supportive-Opposed)
