# Supplementary material for: Stakeholder analysis of the Programme for Improving Mental health carE (PRIME): baseline findings
Source: Int J Ment Health Syst. 2015 Jul 8;9:27. doi: 10.1186/s13033-015-0020-z (PMC4493963; doi:10.1186/s13033-015-0020-z)
Supplement: Additional file 7: — Table S7. Academics: Cross-country stakeholder characteristics regarding the scale-up of mental health care. Country Key: ET – Ethiopia; IN – India; NP – Nepal; SA – South Africa; UG – Uganda (ranked High-Low; Supportive-Opposed or NonMob – Not yet mobilised). [file 13033_2015_20_MOESM7_ESM.docx]

| **TABLE S7: ACADEMICS - CROSS-COUNTRY STAKEHOLDER CHARACTERISTICS REGARDING THE SCALE-UP OF MENTAL HEALTH CARE** | | | | | |
| --- | --- | --- | --- | --- | --- |
| **Stakeholder** | **Involvement in the Issue** | **Interest in the Issue (low, medium, high)** | **Influence/power (low, medium, high)** | **Position**  **(supportive, opposed, non-mobilised)** | **Impact of Issue on Actor (low, medium, high)** |
| Universities | Universities are involved in teaching, producing and disseminating research evidence, and can perform a role in terms of using PRIME as a case study and integrating public mental health into their curricula | IN – High  NP – High  SA – Med  UG – Med  ET - Low/Med | ET – High  IN – Med  SA – Med  UG – Med  NP – Low | ET – Support  NP – Support  UG – Support  IN – NonMob | ET – High  SA – High  UG – Med  IN – Med |
| Research Institutions | Research Institutions are involved in producing and disseminating research evidence and can perform a role in integrating mental health into their research agenda | NP – High  SA – Med  UG – Low  IN – Low | IN – Med  SA – Med  UG – Low  NP – Low | NP – Supportive  IN – NonMob  UG - NonMob | SA – High  NP – Med/High  IN – Low  UG - Low |

Country Key: ET – Ethiopia; IN – India; NP – Nepal; SA – South Africa; UG – Uganda (ranked High-Low; Supportive-Opposed
